# Supplementary material for: Causal interplay between lactose intolerance and gut microbiota: a combined bidirectional Mendelian randomization and in vivo validation study
Source: Front Nutr. 2026 Jun 1;13:1803337. doi: 10.3389/fnut.2026.1803337 (PMC13265576; doi:10.3389/fnut.2026.1803337)
Supplement: Supplementary file 1 [file Data_Sheet_1.zip › supplementary materials/Forward/forest plot/ebi-a-GCST90027699.finngen_R12_E4_LACTONAS.pdf]

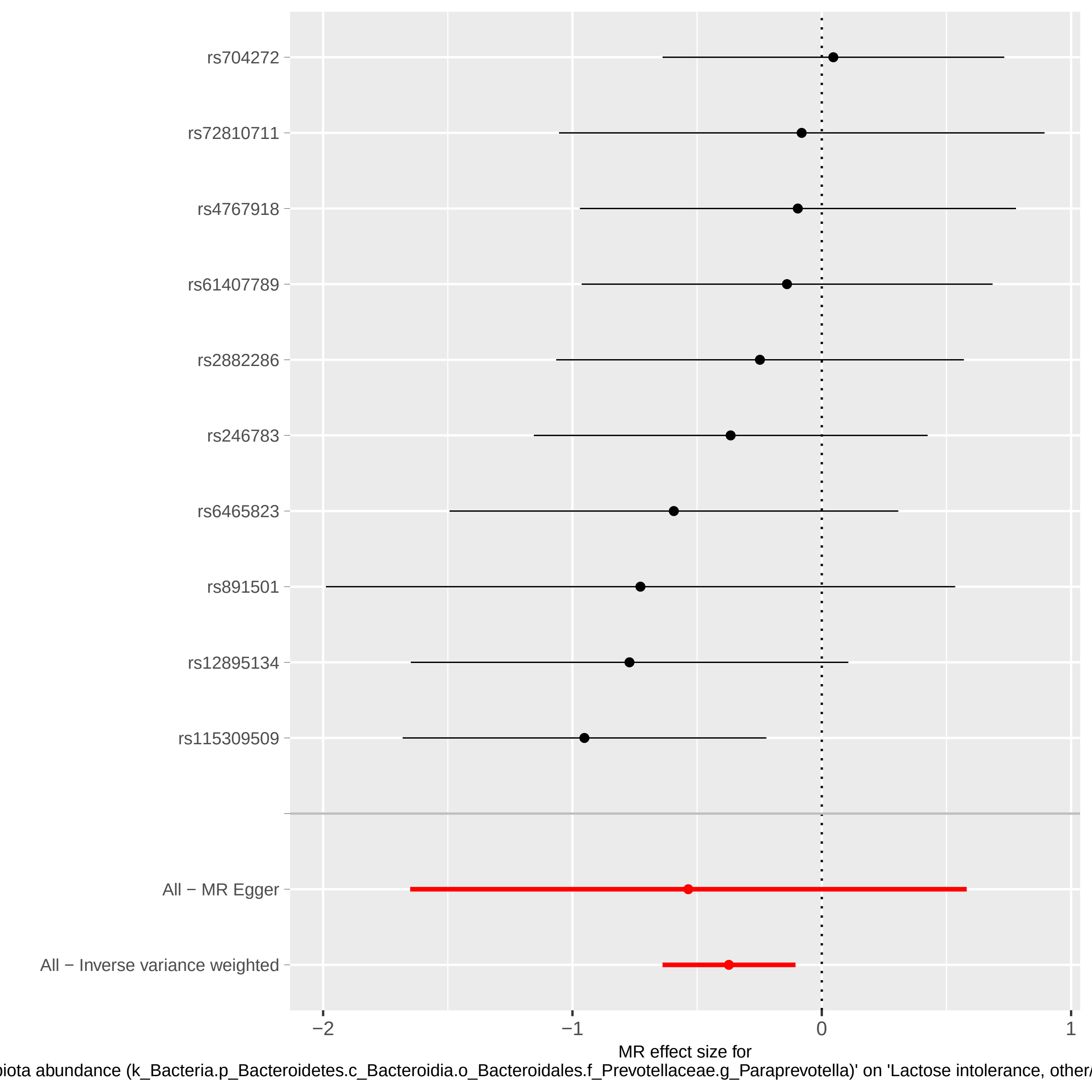

rs704272
rs72810711
rs4767918
rs61407789
rs2882286
rs246783
rs6465823
rs891501
rs12895134
rs115309509
All − MR Egger
All − Inverse variance weighted
−2
−1
0
1
MR effect size for
iota abundance (k_Bacteria.p_Bacteroidetes.c_Bacteroidia.o_Bacteroidales.f_Prevotellaceae.g_Paraprevotella)' on 'Lactose intolerance, other/
